# Syndrome of inappropriate secretion of antidiuretic hormone (SIADH) associated with lateral medullary syndrome: case report and literature review

**DOI:** 10.1186/s12883-016-0641-0

**Published:** 2016-07-27

**Authors:** Hiroro Nakano, Daisuke Yanase, Masahito Yamada

**Affiliations:** 1 Department of Neurology, Kouseiren Takaoka Hospital, Takaoka, Japan; 2 Department of Neurology and Neurobiology of Aging, Kanazawa University Graduate School of Medical Sciences, 13-1 Takara-machi, Kanazawa, 920-8640 Japan

**Keywords:** Syndrome of inappropriate secretion of antidiuretic hormone, Lateral medullary syndrome, Antidiuretic hormone, The nucleus of the solitary tract, The paraventricular nucleus in the hypothalamus

## Abstract

**Background:**

Only one case of syndrome of inappropriate secretion of antidiuretic hormone with lateral medullary syndrome has been reported so far. We report a case of lateral medullary syndrome showing syndrome of inappropriate secretion of antidiuretic hormone and analyze the pathomechanism underlying its clinical features.

**Case presentation:**

A 67-year-old man was admitted to our hospital for dizziness, dysarthria, and dysphagia. He was diagnosed with lateral medullary syndrome based on the neurological examination and brain magnetic resonance imaging. Horner syndrome was absent. Asymptomatic hyponatremia appeared 9 days after admission and the patient was diagnosed with syndrome of inappropriate secretion of antidiuretic hormone. Fluid restriction and intravenous furosemide injection improved the hyponatremia.

**Conclusion:**

Lateral medullary syndrome could be associated with syndrome of inappropriate secretion of antidiuretic hormone.

## Background

Hyponatremia, defined as condition in which serum sodium is <135 mEq/L, is the most common electrolyte abnormality encountered worldwide [1]. One of the major causes of hyponatremia is syndrome of inappropriate secretion of antidiuretic hormone (SIADH), which is caused by various conditions such as malignancies, pulmonary disorders, medications, and acute central nervous system diseases [2]. A previous study reported hyponatremia in nearly 13.8 % of more than 8,500 cases of stroke patients [3]. However, only one case of SIADH with lateral medullary syndrome has been reported so far [4]. In this report, we present SIADH in a patient with lateral medullary syndrome and discuss the pathomechanism of SIADH.

## Case presentation

**Fig. 1:**
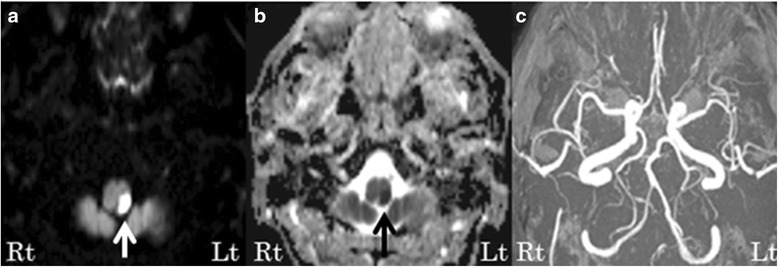
Magnetic resonance imaging (MRI) and MR angiography findings. **a**, **b** Diffusion-weighted magnetic resonance imaging (DWI) showed the hyperintensity in the left dorsolateral medulla (**a**, white arrow) with hypointensity on apparent diffusion coefficient (ADC)-map (**b**, black arrow). **c** MR angiography demonstrated no obvious occlusion

A 67-year-old man with untreated diabetes mellitus developed dizziness, followed by dysarthria and dysphagia, 4 days before admission to our hospital. On admission, he was fully conscious, and the physical examination was almost normal except for hypertension and tachycardia in a regular rhythm. Neurological examination revealed equal and round pupils with a normal light reflex. His extraocular movements were full range without diplopia, but he showed gaze-evoked horizontal nystagmus, dysarthria, and dysphagia. He had no complaint of dysgeusia. Muscle tonus was normal without obvious paralysis in all four limbs. Examination of the sensory system revealed hypothermia and hypoalgesia with normal tactile sensation over the left side of his face and right half of his trunk and limbs. Vibration and position sensation were normal in all four limbs. He presented with ataxia in his left upper and lower limbs. Tendon reflexes were normal in his upper limbs and absent in his lower limbs without the extensor plantar reflex. Brain magnetic resonance imaging (MRI) demonstrated hyperintensity in the left side of the dorsolateral medulla with diffusion weighted imaging (DWI) with hypointensity on apparent diffusion coefficient (ADC)-map (Fig. 1). He was diagnosed as having lateral medullary syndrome and was treated with oral clopidogrel (continued during admission), intravenous argatroban for 3 days, edaravone for 9 days, and hydration with normal saline (1.5 L/day).

Hematological examination showed normal serum sodium levels of 139 mEq/L on admission, however, the serum sodium levels decreased gradually, and 9 days after admission (13 days after onset), he developed asymptomatic hyponatremia (109 mEq/L) with a decreased serum osmolarity of 223 mOsm/L (normal range, 275 – 295 mOsm/L). The turgor in all four limbs was normal, and he did not present with obvious thirst. Thyroid, renal, and adrenal functions were normal, but increased serum levels of antidiuretic hormone (ADH) (6.9 pg/mL; normal range, 0.3 – 4.2 pg/mL) were observed. Urine osmolarity was 668 mOsm/kgH_2_O (normal range, 100 – 1,300 mOsm/kgH_2_O), and urine sodium was 121 mOsm/L (normal range, 40 – 90 mOsm/L). Thoracoabdominal CT images showed no abnormalities, such as mass lesions or lymphadenopathy. He was diagnosed as having SIADH based on the diagnostic criteria of Bartter and Schwartz [5]. He was treated with fluid restriction and intravenous furosemide injections.

Laboratory examination on the 23rd day after admission showed almost normal serum sodium levels (136 mEq/L) with normal serum ADH levels (1.2 pg/mL). His neurological symptoms, such as dizziness and dysarthria, also gradually resolved, and thereafter, hyponatremia was not observed.

## Conclusion

Our patient with lateral medullary syndrome showed asymptomatic hyponatremia 13 days after the onset of lateral medullary syndrome. With the exclusion of other causes of hyponatremia, we diagnosed the hyponatremia as SIADH. It has been reported that SIADH is difficult to distinguish from cerebral salt wasting syndrome, another cause of hyponatremia characterized by renal loss of sodium and decreases in extracellular fluid volume during intracranial disorders [6]; nonetheless, the abnormal ADH secretion and lack of clinical symptoms of dehydration in our patient were consistent with the characteristics of SIADH [7], although precise evaluation of the volemic state has been considered difficult [8]. Thoracoabdominal CT images indicated no evidence of ectopic ADH producing tumors. Nine days after admission, the serum sodium levels gradually improved. After the primary treatment for SIADH, our patient presented almost normal serum sodium levels with resolution of his neurological symptoms, and thereafter, he showed no relapse of hyponatremia, indicating that SIADH could be closely related to ischemic damage in the dorsolateral medulla. As edaravone was administered for 9 days after admission, the possibility that edaravone contributed to the pathophysiology of SIADH cannot be ruled out, although no case of SIADH associated with edaravone has been reported so far.

In retrograde tracing studies, two major neural pathways were identified between the dorsolateral medullary area and the hypothalamus [9, 10]. These include (1) the sympathetic descending tract and (2) the interacting neural pathway between the paraventricular nucleus in the hypothalamus (PVH) and the nucleus of the solitary tract (NTS), although the neurological function of the later pathway is not understood yet. The NTS could be involved in the mechanism of SIADH in lateral medullary syndrome, as discussed in the previously reported case [4]. However, as the NTS is occasionally involved in lateral medullary syndrome [11], the ischemic damage of the NTS cannot simply explain the pathogenesis of SIADH in lateral medullary syndrome. Although SIADH in our patient would be related to the exaggerated ADH response to ischemic damage as discussed in the previously reported [12], we speculated that the ischemic damage of the ascending neural pathway from the NTS to the PVH could be related to the pathogenesis of SIADH in lateral medullary syndrome.

We analyzed the clinical findings on lateral medullary syndrome and SIADH, from our patient and the previously reported one [4], and compared them with the typical features of lateral medullary syndrome [13, 14] (Table 1). The three clinical symptoms, including (1) crossed sensory disturbance, (2) bulbar palsy, and (3) cerebellar ataxia, are considered the main symptoms of lateral medullary syndrome [13, 14], and both patients showed these typical symptoms. Interestingly, asymptomatic hyponatremia was identified at 13 days in our patient and at 15 days in the previously reported patient after the onset of lateral medullary syndrome. The main difference in the clinical features between our patient and the previously reported one was the presence of Horner syndrome. The absence of Horner syndrome in our patient indicated that the sympathetic descending tract was spared from severe ischemic damages, and the sympathetic descending tract would be anatomically separated from the ascending neural pathway from the NTS to PVH. Both patients presented no sign of dysgeusia, suggesting that the NTS was spared from severe ischemic damages, although the MRI findings in both patients were similar to those of typical lateral medullary syndrome [14]. The ascending neural pathway from the NTS to PVH may be associated with ADH secretion, and this pathway may be less involved in most patients with lateral medullary syndrome, however, the ascending neural pathway could be more involved in the clinical manifestations observed in our patient and the previously reported one [4]. It is suggested that the minority of patients with lateral medullary syndrome would present with damage of the ascending neural pathway from the NTS to PVH.

**Table 1:** Clinical features of SIADH in patients with Wallenberg syndrome [4, 11, 12]

	This case	Previous case [4]	Typical features [11, 12]
Age(y.o.)/Sex	67/Male	44/Male	mean age: 57 ± 11.9
			Male: Female = 2.25:1
Initial symptoms	dizziness, dysarthria, dysphagia	headache, dysphagia, hiccups,	dizziness, vertigo, gait ataxia, dysarthria, dysphagia
Neurological symptoms	crossed sensory disturbance, cerebellar ataxia, nystagmus	crossed sensory disturbance, cerebellar ataxia	crossed sensory disturbance, cerebellar ataxia
Horner syndrome	-	+	+
Dysgeusia	-	-	occasionally observed
Past history	diabetes mellitus	not described	hypertension, diabetes mellitus, cigarette smoking
Treatment for cerebral infarction	edaravone oral clopidogrel argatroban	edaravone	varies depend on the causes
Interval days between the onset of Wallenberg syndrome and hyponatremia	thirteen days	fifteen days	
Symptom of hyponatremia	asymptomatic	asymptomatic	
Treatment for hyponetremia	restriction of fluid intake, intravenous furosemide	restriction of fluid intake	
Clinical course	improved	improved	

We described SIADH in a patient with lateral medullary syndrome. Taken together with the previously reported case, we suggest that lateral medullary syndrome could be associated with hyponatremia.

## Abbreviations

ADC, apparent diffusion coefficient; ADH, antidiuretic hormone; DWI, diffusion weighted imaging; NTS, the nucleus of the solitary tract; PVH, the paraventricular nucleus in the hypothalamus; SIADH, syndrome of inappropriate secretion of antidiuretic hormone
