# Erratum to: Syndrome of inappropriate secretion of antidiuretic hormone (SIADH) associated with lateral medullary syndrome: case report and literature review

**DOI:** 10.1186/s12883-016-0713-1

**Published:** 2016-10-03

**Authors:** Hiroto Nakano, Daisuke Yanase, Masahito Yamada

**Affiliations:** 1 Department of Neurology, Kouseiren Takaoka Hospital, Takaoka, Japan; 2 Department of Neurology and Neurobiology of Aging, Kanazawa University Graduate School of Medical Sciences, 13-1 Takara-machi, Kanazawa, 920-8640 Japan

## Erratum

After publication of the original article [1], it came to the authors’ attention that the given name of the first author was spelled wrongly, due to a misinterpreted author correction during proofing. The first author’s name (Hiroto Nakano) is published correctly in this erratum.
